# Supplementary material for: High Throughput Identification of Novel Conotoxins from the Vermivorous Oak Cone Snail (Conus quercinus) by Transcriptome Sequencing
Source: Int J Mol Sci. 2018 Dec 5;19(12):3901. doi: 10.3390/ijms19123901 (PMC6321112; doi:10.3390/ijms19123901)
Supplement: Supplementary file 1 [file ijms-19-03901-s001.zip › ijms-391730-supplementary-final/ijms-391730-supplementary Figure S1-S5.pdf]

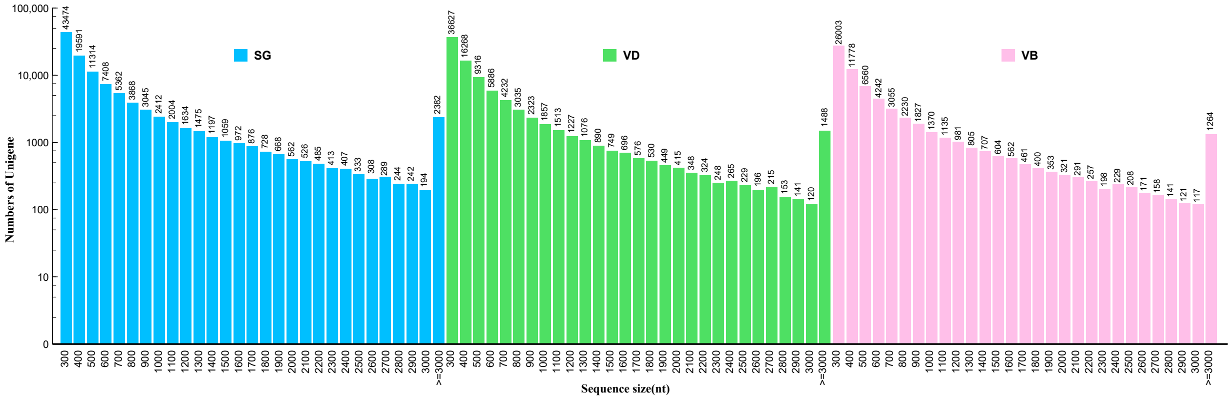


**Supplementary Figure S1.** Length distribution of unigenes for venom duct (VD), salivary gland (SG) and venom bulb (VB).

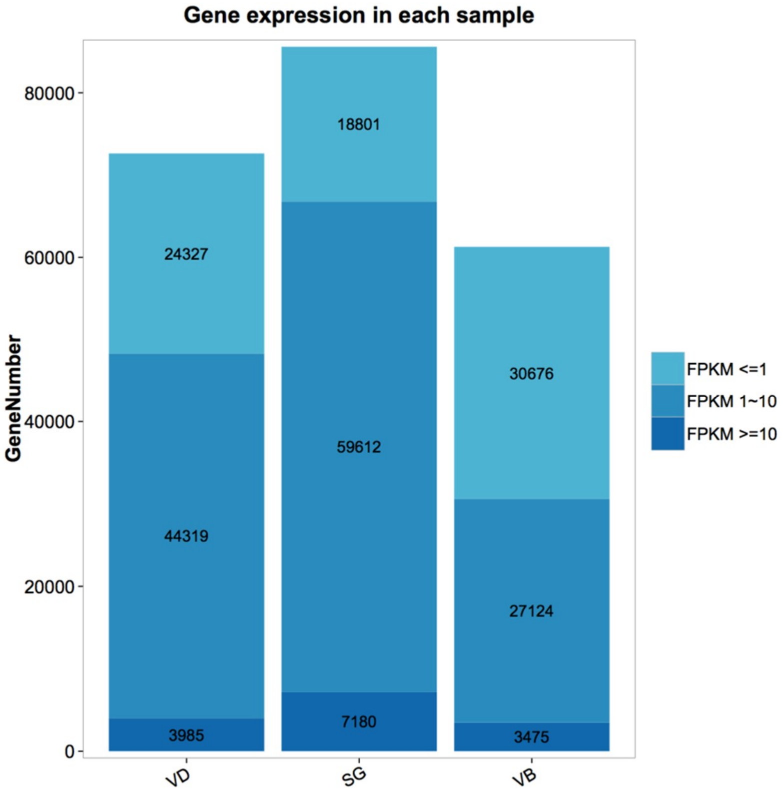


**Supplementary Figure S2.** Distribution of gene transcription levels in venom duct (VD), salivary gland (SG) and venom bulb (VB).

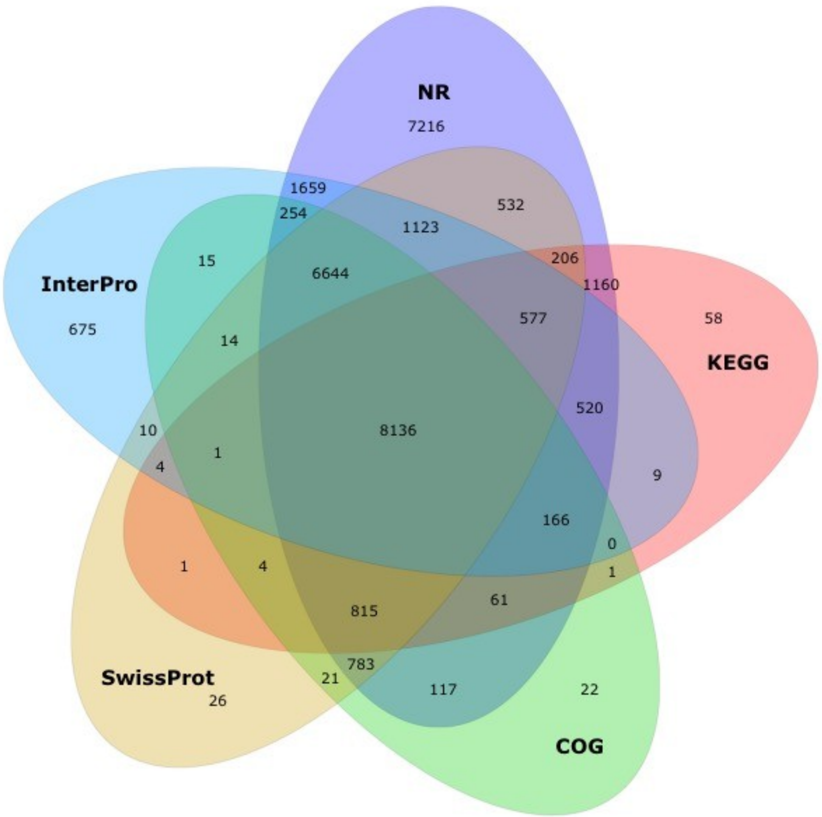


**Supplementary Figure S3.** Functional annotation of all assembled genes in venom duct (VD), salivary gland (SG) and venom bulb (VB) based on multiple public databases.

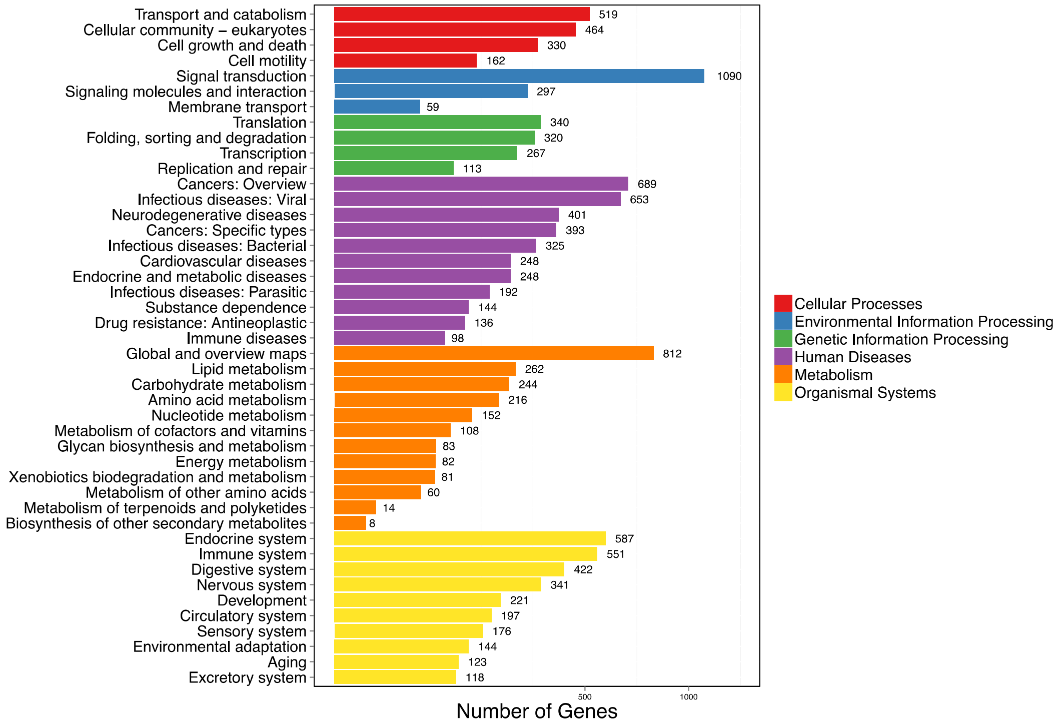


**Supplementary Figure S4.** Enriched KEGG pathways and number distribution of annotated genes in each pathway.

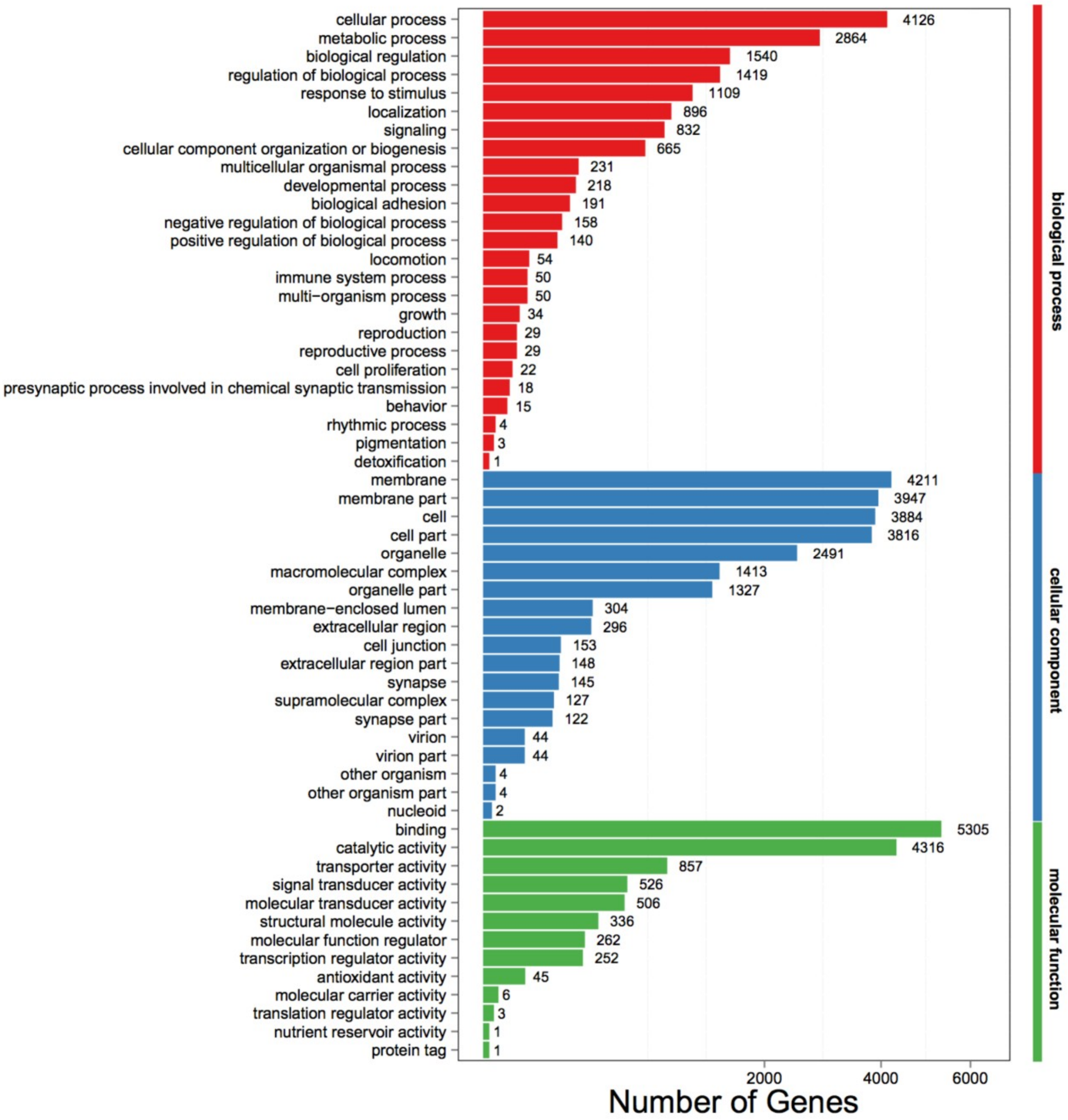


**Supplementary Figure S5.** Enriched GO terms and number distribution of annotated genes in each term.
